# Neoadjuvant disitamab vedotin (RC-48) plus a PD-1 inhibitor in muscle-invasive bladder cancer: a multicenter real-world study with stratification by HER2 expression and tertiary lymphoid structures

**DOI:** 10.3389/fimmu.2026.1891151

**Published:** 2026-09-28

**Authors:** Xue Peng Rao, Xin Chang Zou, Xiang Liu, Lie Yu Xu, Jian Biao Huang, Yu Yang Yuan, Tao Chen, Tao Zeng

**Affiliations:** 1 Department of Urology, Second Affiliated Hospital of Nanchang University, Jiangxi Medical College, Nanchang, China; 2 Department of Urology, Jiangxi Provincial People’s Hospital, Nanchang, China; 3 Jiangxi Cancer Hospital & Institute, Jiangxi Clinical Research Center for Cancer, The Second Affiliated Hospital of Nanchang Medical College, Nanchang, China; 4 Department of Urology, First Affiliated Hospital of Nanchang University, Nanchang, China

**Keywords:** disitamab vedotin, HER2, multicenter real-world study, muscle-invasive bladder cancer, neoadjuvant therapy, PD-1 inhibitor, tertiary lymphoid structure

## Abstract

**Objective:**

To assess the efficacy and safety of neoadjuvant RC-48 combined with a PD-1 inhibitor in patients with muscle-invasive bladder cancer (MIBC), and to investigate the stratification utility of HER2 expression and tumor-associated tertiary lymphoid structures (TLS) as predictive variables for treatment response and clinical prognosis.

**Patients and methods:**

This multicenter retrospective real-world study enrolled 38 patients with MIBC from the First Affiliated Hospital of Nanchang University, the Second Affiliated Hospital of Nanchang University, Jiangxi Cancer Hospital, and Jiangxi Provincial People’s Hospital. All patients received at least four cycles of neoadjuvant therapy comprising RC-48 combined with a PD-1 inhibitor, followed by radical cystectomy (RC). The TLS in the pre-treatment specimens was evaluated using hematoxylin-eosin (HE) staining, and the expression level of HER2 was assessed by immunohistochemistry (IHC) method. The primary endpoint was the pathological complete response (pCR) rate, and secondary endpoints included radiographic complete response (CR) rate, disease-free survival (DFS), and overall survival (OS).

**Results:**

Among the 38 patients, the pCR rate was 34.2%, and the overall CR rate by radiographic assessment was 36.8%, with a PR rate of 31.6%. After a median follow-up of 18.0 months (range, 9–29 months), the 12-month DFS rate was 89.4% (95% CI: 74.2%–95.9%), and the 12-month OS rate was 100% (95% CI: 100%–100%). HER2 overexpression was significantly associated with a higher objective response rate (ORR) (OR = 22.50, 95% CI: 1.51 - 335.33, p = 0.024). In stratified analyses based on TLS, both TLS-2 (primary lymphoid follicles) and TLS-3 (secondary lymphoid follicles with germinal centers) demonstrated significant associations with higher ORR, with ORs of 20.00 (95% CI: 2.75 - 145.48, p = 0.003) and 36.00 (95% CI: 2.72 - 476.21, p = 0.007), respectively, suggesting that greater TLS with enhanced therapeutic benefit. Furthermore, N1 stage (OR = 0.06, 95% CI: 0.01 - 0.59, p = 0.016), N2 stage (OR = 0.04, 95% CI: 0.00 - 0.62, p = 0.021), and T3 stage (OR = 0.08, 95% CI: 0.01 - 0.46, p = 0.004) were each significantly associated with poorer treatment response. Treatment-related adverse events (TRAEs) were generally manageable.

**Conclusions:**

The combination of RC-48 and a PD-1 inhibitor exhibits promising efficacy as a neoadjuvant therapy for MIBC. Both HER2 expression and TLS maturity serve as effective stratification indicators for treatment response and prognosis. These findings support further evaluation of this combined regimen in biomarker-driven prospective trials.

## Introduction

Muscle-invasive bladder cancer (MIBC) is a common aggressive malignancy of the urinary system, and radical cystectomy combined with neoadjuvant therapy remains the standard treatment modality (1, 2). Although cisplatin-based neoadjuvant chemotherapy can improve patient outcomes, its clinical application faces several challenges (3). Approximately 30%–50% of patients are unable to tolerate cisplatin-based regimens due to advanced age, renal insufficiency, or comorbidities. Moreover, even after completing the full course of chemotherapy, about 40%–50% of patients experience disease recurrence or metastasis within five years after surgery (4, 5). Therefore, exploring novel neoadjuvant treatment strategies with better efficacy and broader applicability has become a critical breakthrough point for advancing therapeutic standards in this field.

In recent years, the emergence of immunotherapy has significantly changed the treatment landscape of MIBC (6). Immune checkpoint inhibitors, represented by programmed death receptor-1 and its ligand inhibitors, have shown unique advantages in neoadjuvant therapy by activating the body’s own antitumor immune response (7, 8). Multiple single-arm studies have reported pathological complete response rates of 30%-40% with immunotherapy monotherapy, along with a generally favorable safety profile. However, the efficacy of immunotherapy monotherapy is heterogeneous, and not all patients derive benefit (9, 10). Meanwhile, although immunotherapy combined with chemotherapy has improved pathological response rates to some extent, the incremental benefit compared with traditional chemotherapy remains limited, and the associated treatment-related toxicities require careful evaluation (11). How to enhance therapeutic efficacy while ensuring safety and broad applicability remains a key direction that warrants in-depth exploration in current clinical research.

The emergence of antibody-drug conjugates (ADCs) has provided novel synergistic strategies for combination immunotherapy (12). RC48 exhibits a dual mechanism of action combining targeted precision killing with immune activation (13). This drug delivers cytotoxic agents precisely into tumor cells via antibodies, which not only effectively kills tumor cells but also induces immunogenic cell death and releases tumor antigens, thereby creating synergistic effects with immune checkpoint inhibitors (14). In the treatment of advanced urothelial carcinoma, multiple clinical studies of RC48 monotherapy and its combination with immunotherapy have demonstrated encouraging antitumor activity and manageable safety profiles, laying a solid foundation for extending its use to earlier disease stages such as neoadjuvant therapy (15, 16).

Based on this, we conducted a multicenter real-world study to evaluate the efficacy and safety of RC48 combined with PD-1 inhibitors as neoadjuvant therapy for muscle-invasive bladder cancer. Unlike clinical trials, real-world studies better reflect patient heterogeneity and the complexity of diagnosis and treatment in clinical practice. This study will also perform stratified analyses based on HER2 expression levels and TLS to explore efficacy differences among patients with different biological characteristics, aiming to provide references for clinically identifying potential beneficiary populations.

## Patients and methods

### Patients

This study retrospectively analyzed patients with MIBC who received neoadjuvant therapy consisting of RC-48 in combination with PD-1 inhibitors between June 2022 and January 2025 at the First Affiliated Hospital of Nanchang University, the Second Affiliated Hospital of Nanchang University, Jiangxi Cancer Hospital, and Jiangxi Provincial People’s Hospital. The study protocol was reviewed and approved by the ethics committees of all participating institutions, in accordance with the ethical principles outlined in the Declaration of Helsinki. Due to the retrospective nature of the study and exclusive use of anonymized clinical data, the ethics committees waived the requirement for obtaining patient informed consent. Patients were included based on the following criteria: (1) a histopathological diagnosis of MIBC with clinical stage cT2-4aN0-2M0; (2) completion of at least four cycles of RC-48 combined with a PD-1 inhibitor as neoadjuvant therapy; (3) undergoing radical cystectomy following neoadjuvant treatment. Exclusion criteria included: (1) prior systemic therapy for bladder cancer (e.g., chemotherapy, immunotherapy, or targeted therapy); (2) evidence of distant metastasis (cM1); (3) absence of key clinicopathological data or inability to assess critical variables; (4) concurrent presence of other active malignancies. See **Supplementary Figure S1** for the patient screening flowchart.

### Therapeutic approach

All patients received neoadjuvant therapy consisting of RC-48 combined with a PD-1 inhibitor. RC-48 was administered intravenously at a dosage of 2.0 mg/kg every two weeks, while the PD-1 inhibitors, either Toripalimab (240 mg) or Tislelizumab (200 mg), were administered intravenously every three weeks. The treatment was continued until the scheduled surgery, but could be discontinued earlier in cases of disease progression, unacceptable toxicity, or at the patient’s request.

### Assessment of HER2 and TLS

HER2 expression status was evaluated using IHC on specimens obtained from transurethral resection of bladder tumors prior to treatment. IHC scoring was independently performed by two senior pathologists according to the Expert Consensus on HER2 Detection in Urological Tumors of China (2022 Edition), and was categorized into four grades: IHC 0, IHC 1+, IHC 2+, and IHC 3+. Any discrepancies between the two pathologists were resolved by review and consensus from a third senior pathologist. Concurrently, the presence and classification of tertiary lymphoid structures (TLS) were assessed on H&E-stained full-section slides derived from the same surgical resection specimens. All evaluations were performed independently by two experienced pathologists who were blinded to clinical outcomes and treatment allocation; in cases of disagreement, a third senior pathologist served as adjudicator to reach a final consensus. The minimum criterion for TLS identification was defined as the presence of at least one aggregate containing ≥20 lymphoid cells with discernible architectural organization. Based on the degree of lymphocyte aggregation and maturation, TLS were further classified into three subtypes: early lymphocyte aggregation (TLS-1), characterized by loose lymphocyte clusters without tissue organization; primary lymphoid follicles (TLS-2), defined as lymphocyte aggregates with primary follicular structure but lacking germinal centers; and secondary lymphoid follicles (TLS-3), characterized by mature follicular structure with well-developed germinal centers.

### Efficacy evaluation and prognostic analysis

Patient baseline characteristics, including age, gender, and Eastern Cooperative Oncology Group (ECOG) performance status, were retrospectively collected. Clinicopathological data comprised clinical T and N staging, histologic subtype, HER2 expression status, and tertiary lymphoid structure classification. Treatment-related details included the number of cycles of RC-48 and PD-1 inhibitors administered. Follow-up data were also gathered. After completing neoadjuvant therapy, all patients underwent RC. Postoperative specimens were meticulously examined by pathologists to document the ypT stage, ypN stage, surgical margin status, and tumor regression grade of the primary lesion, with particular attention to the proportion of residual tumor cells. The primary efficacy endpoint of this study was the pCR rate, defined as the absence of residual tumor cells in both the primary tumor and regional lymph nodes of radical cystectomy specimens. Radiographic complete response was defined as the complete disappearance of all target lesions on cross-sectional imaging and the absence of new lesions, as assessed by Response Evaluation Criteria in Solid Tumors version 1.1. Partial response (PR) is defined as at least a 30% decrease in the sum of the longest diameters of all target lesions compared with baseline, lasting for at least 4 weeks. Secondary efficacy endpoints included DFS, defined as the time from treatment initiation to the first occurrence of disease recurrence, progression, or death from any cause, and OS, defined as the time from treatment initiation to death from any cause. Follow-up assessments were conducted through outpatient visits, hospitalization records, and telephone interviews, with a data cutoff date set for February 2026.

### Safety evaluation

TRAEs were systematically recorded through active medical record reviews and patient follow-ups, with grading and classification performed in accordance with the National Cancer Institute’s Common Terminology Criteria for Adverse Events (CTCAE) version 5.0. All grades of TRAEs were documented, with specific emphasis on severe adverse events categorized as Grade 3 or higher. Prior to each treatment cycle, patients underwent comprehensive evaluations, including complete blood counts, liver and kidney function tests, thyroid function assessments, and cardiac enzyme analyses, to proactively monitor for potential adverse events.

### Statistical analysis

Statistical analyses were conducted using R software (version 4.3.2) and SPSS version 27.0. Descriptive statistics were presented as medians with interquartile ranges for continuous variables and as frequencies with percentages for categorical variables. Univariate logistic regression analysis was utilized to evaluate the relationships between HER2 expression status, TLS, clinical stage, and imaging-based response rates. Survival outcomes, including DFS and OS, were analyzed using the Kaplan-Meier method, with intergroup comparisons performed via the log-rank test. Stratified survival analyses were conducted according to HER2 expression status and TLS status. All statistical tests were two-sided, with a significance threshold of P < 0.05.

## Results

### Patient clinical and pathological features

A total of 38 patients with MIBC who received neoadjuvant therapy were enrolled, all of whom underwent radical cystectomy following treatment. The median age was 65 years (Q_1_–Q_3_: 56–70), and 68.42% (26 cases) were male. ECOG performance status was 0 in 65.79% (25 cases) and 1 in 34.21% (13 cases). The most common histological subtype was pure urothelial carcinoma (60.53%, 23 cases), followed by squamous differentiation (21.05%, 8 cases) and other differentiation types (18.42%, 7 cases). Lymphovascular invasion was positive in 36.84% (14 cases). Clinical staging was predominantly T2 (63.16%, 24 cases), with T3 and T4 stages accounting for 28.95% (11 cases) and 7.89% (3 cases), respectively. Nodal staging showed N0 in 44.74% (17 cases), N1 in 42.11% (16 cases), and N2 in 13.16% (5 cases). Regarding treatment regimens, RC48 combined with toripalimab was administered in 55.26% (21 cases), and combined with tislelizumab in 44.74% (17 cases). The distribution of HER2 expression before neoadjuvant therapy was as follows: 3+ in 42.11% (16 cases), 2+ in 23.68% (9 cases), 1+ in 21.05% (8 cases), and 0 in 13.16% (5 cases). In terms of TLS stratification, the primary lymphoid follicle type was the most common (47.37%, 18 cases), while early lymphocyte aggregation and secondary lymphoid follicle type each accounted for 26.32% (10 cases each). Detailed baseline characteristics are presented in **Table 1**. The median interval between the last treatment and surgery was 25 days (range, 21–28 days). All patients achieved R0 resection (100%), and postoperative complications were predominantly Clavien-Dindo grade 0-I (73.7%), with an adjuvant therapy rate of 15.8%. Detailed data are presented in **Supplementary Table S4**.

**Table 1:** Baseline characteristics of patients.

Variables	Cases/values
Age, M (Q_1_, Q_3_)	65 (56, 70)
Gender, n(%)	
Female	12 (31.58)
Male	26 (68.42)
ECOG, n(%)	
0	25 (65.79)
1	13 (34.21)
Organizational subtype variation, n(%)	
Pure urothelial carcinoma	23 (60.53)
Urothelial carcinoma with squamous differentiation	8 (21.05)
Urothelial carcinoma with other differentiation	7 (18.42)
Lymphovascular invasion, n(%)	
No	24 (63.16)
Yes	14 (36.84)
T stage, n(%)	
T2	24 (63.16)
T3	11 (28.95)
T4	3 (7.89)
N stage, n(%)	
N0	17 (44.74)
N1	16 (42.11)
N2	5 (13.16)
Drug type, n(%)	
RC48 + toripalimab	21 (55.26)
RC48 + tislelizumab	17 (44.74)
HER2, n(%)	
0	5 (13.16)
1+	8 (21.05)
2+	9 (23.68)
3+	16 (42.11)
TLS, n(%)	
Early lymphoid aggregation	10 (26.32)
Primary lymphoid follicle	18 (47.37)
Secondary lymphoid follicle	10 (26.32)

ECOG, eastern Cooperative Oncology Group performance status. Urothelial carcinoma with other differentiation: including glandular differentiation, micropapillary differentiation, etc.

### Evaluation of treatment efficacy

Among the 38 patients who received neoadjuvant therapy, the radiographic CR rate was 36.8%, the pCR rate was 34.2%, the partial response (PR) rate was 31.6% (12 cases), 10 cases had stable disease (SD), and 2 cases showed disease progression (PD). With a median follow-up of 18.0 months (range: 9–29 months), the 12-month DFS rate was 89.4% (95% CI: 74.2%–95.9%), and the 12-month OS rate was 100% (95% CI: 100%–100%). The corresponding Kaplan-Meier survival curves are presented in **Figure 1**.

**Figure 1:**
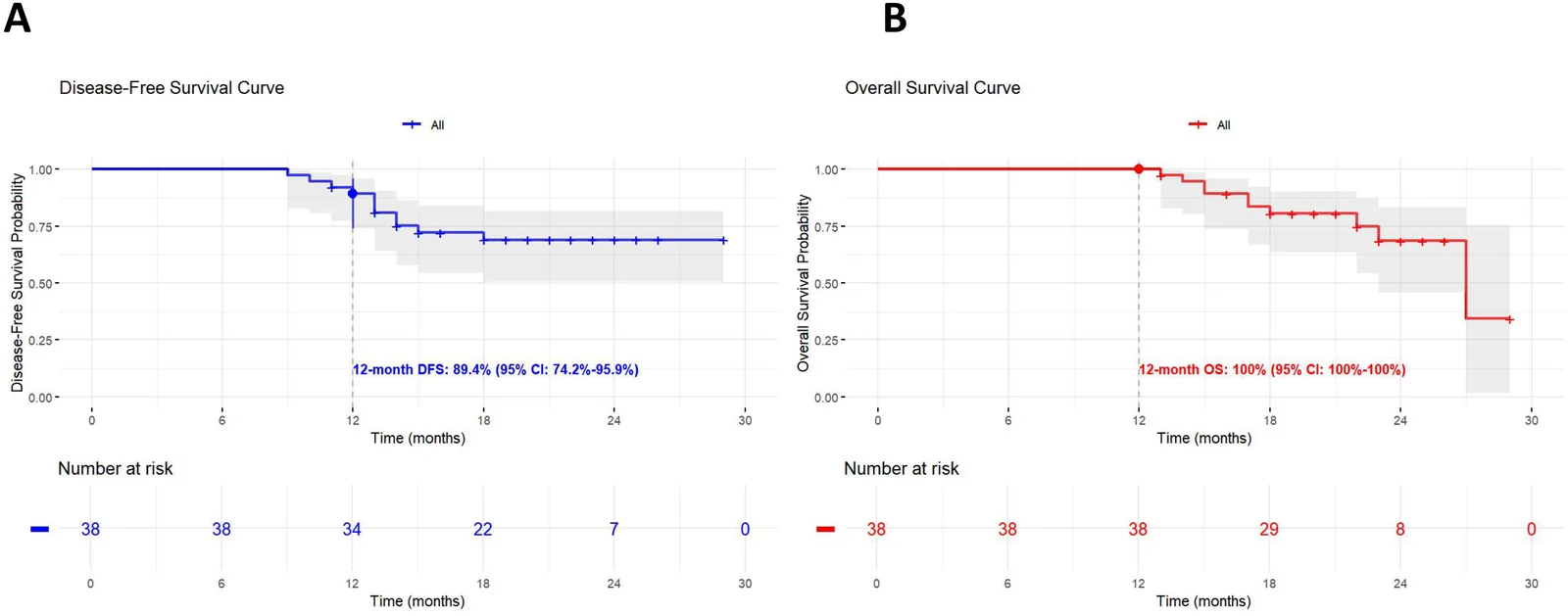
Analysis of DFS **(A)** and OS **(B)** for all patients.

**Table 2** provides the univariate logistic regression analysis results for factors influencing the objective response rate (ORR, defined as CR + PR), comparing responders (n = 26) and non-responders (n = 12). The analysis revealed that most baseline characteristics, such as age (≤60 years vs. >60 years), gender, ECOG performance status (0 vs. 1), and treatment regimen (RC48 combined with toripalimab vs. RC48 combined with tislelizumab), were not significantly correlated with ORR. However, certain pathological and clinical variables showed notable associations. Specifically, patients with squamous differentiation (OR = 0.09, 95% CI: 0.01–0.59, P = 0.012) or other differentiation subtypes (OR = 0.11, 95% CI: 0.02–0.77, P = 0.026) were significantly less likely to achieve remission compared to those with pure urothelial carcinoma. Positive lymphovascular invasion was similarly associated with reduced remission rates (OR = 0.15, 95% CI: 0.03–0.68, P = 0.014). Furthermore, clinical staging analysis indicated significantly lower response rates for patients with T3 stage (OR = 0.08, 95% CI: 0.01–0.46, P = 0.004) and T4 stage (OR = 0.07, 95% CI: 0.00–1.05, P = 0.054) in comparison to those with T2 stage tumors. Patients with N1 stage (OR = 0.06, 95% CI: 0.01–0.59, P = 0.016) and N2 stage (OR = 0.04, 95% CI: 0.00–0.62, P = 0.021) also demonstrated significantly reduced response rates relative to those with N0 stage disease. Notably, HER2 expression analysis revealed that patients with IHC 3+ had a significantly increased likelihood of achieving remission (OR = 22.50, 95% CI: 1.51–335.33, P = 0.024). Additionally, TLS stratification highlighted that patients with primary lymphoid follicle type (OR = 20.00, 95% CI: 2.75–145.48, P = 0.003) and secondary lymphoid follicle type (OR = 36.00, 95% CI: 2.72–476.21, P = 0.007) showed substantially higher response rates compared to those with early lymphocyte aggregation. Patient pathologic downstaging is shown in **Supplementary Figure S3**.

**Table 2:** Results of univariate logistic regression analysis of clinicopathological variables and objective response rate.

Variables	β	S.E	Z	*P*	OR (95%CI)
Age					
≤60					Reference
>60	0.47	0.70	0.67	0.504	1.60 (0.40 ~ 6.36)
Gender, n(%)					
Female					Reference
Male	0.12	0.75	0.16	0.874	1.12 (0.26 ~ 4.85)
ECOG, n(%)					
0					Reference
1	0.63	0.78	0.81	0.420	1.87 (0.41 ~ 8.63)
Histological differentiation, n(%)					
Pure urothelial carcinoma					Reference
Urothelial carcinoma with squamous differentiation	-2.41	0.96	-2.52	0.012	0.09 (0.01 ~ 0.59)
Urothelial carcinoma with other differentiation	-2.18	0.98	-2.22	0.026	0.11 (0.02 ~ 0.77)
Lymphovascular invasion, n(%)					
No					Reference
Yes	-1.90	0.77	-2.47	0.014	0.15 (0.03 ~ 0.68)
T stage, n(%)					
T2					Reference
T3	-2.51	0.88	-2.85	0.004	0.08 (0.01 ~ 0.46)
T4	-2.64	1.37	-1.92	0.054	0.07 (0.00 ~ 1.05)
N stage, n(%)					
N0					Reference
N1	-2.77	1.15	-2.42	0.016	0.06 (0.01 ~ 0.59)
N2	-3.18	1.38	-2.31	0.021	0.04 (0.00 ~ 0.62)
Drug type, n(%)					
RC48 + toripalimab					Reference
RC48 + tislelizumab	0.18	0.71	0.26	0.796	1.20 (0.30 ~ 4.78)
HER2, n(%)					
0					Reference
1+	-1.54	1.41	-1.10	0.273	0.21 (0.01 ~ 3.37)
2+	2.48	1.40	1.78	0.076	12.00 (0.77 ~ 186.36)
3+	3.11	1.38	2.26	0.024	22.50 (1.51 ~ 335.33)
TLS, n(%)					
Early lymphoid aggregation					Reference
Primary lymphoid follicle	3.00	1.01	2.96	0.003	20.00 (2.75 ~ 145.48)
Secondary lymphoid follicle	3.58	1.32	2.72	0.007	36.00 (2.72 ~ 476.21)

### Prognostic stratification analysis

To further assess the influence of various clinicopathological factors on patient prognosis, Kaplan-Meier survival analyses were performed based on the variables significantly associated with treatment response identified in the univariate logistic regression analysis. The results revealed that patients with squamous differentiation or other differentiation subtypes had significantly worse DFS and OS compared to those with pure urothelial carcinoma (both P < 0.001). Similarly, the presence of lymphovascular invasion was strongly associated with poorer prognosis (DFS: P < 0.001; OS: P = 0.001). Patients with advanced T-stage (T3–T4) and N-stage (N1–N2) demonstrated significantly inferior survival outcomes compared to those with T2-stage and N0-stage disease (all P < 0.05). Notably, HER2 overexpression was associated with improved DFS and OS (DFS: P = 0.007; OS: P = 0.012). Additionally, patients with the higher TLS subtype exhibited significantly better survival outcomes compared to those with the early clustered subtype (DFS: P = 0.003; OS: P = 0.042) (**Figures 2**, **3**). The subgroup analysis of drug types based on DFS and OS is presented in **Supplementary Tables S1** and **S2**. **Supplementary Figure S2** indicates that different PD-1 drug types do not lead to prognostic differences.

**Figure 2:**
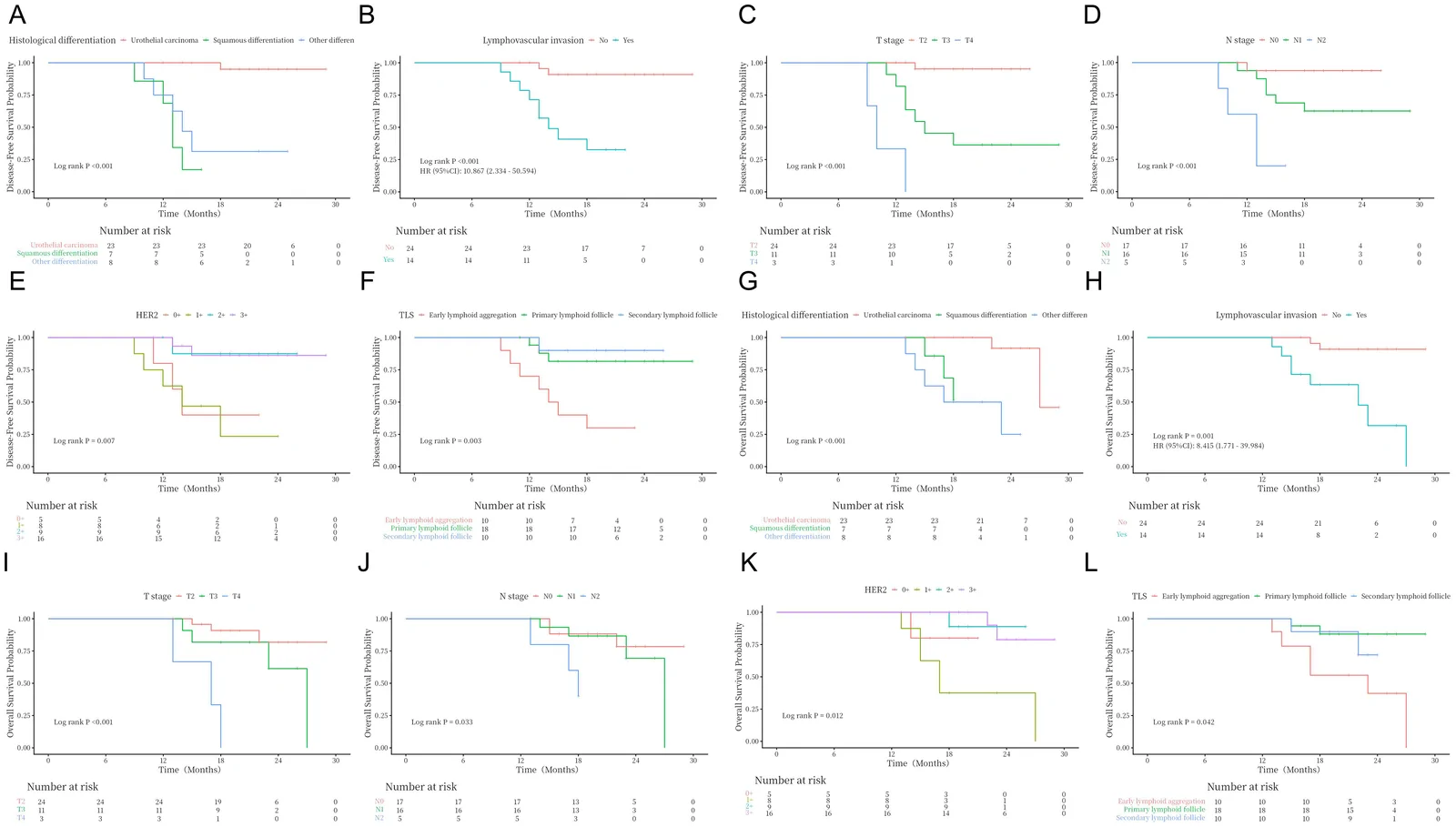
Stratification of clinical and pathological variables in DFS **(A-F)** and OS **(G-L)**.

**Figure 3:**
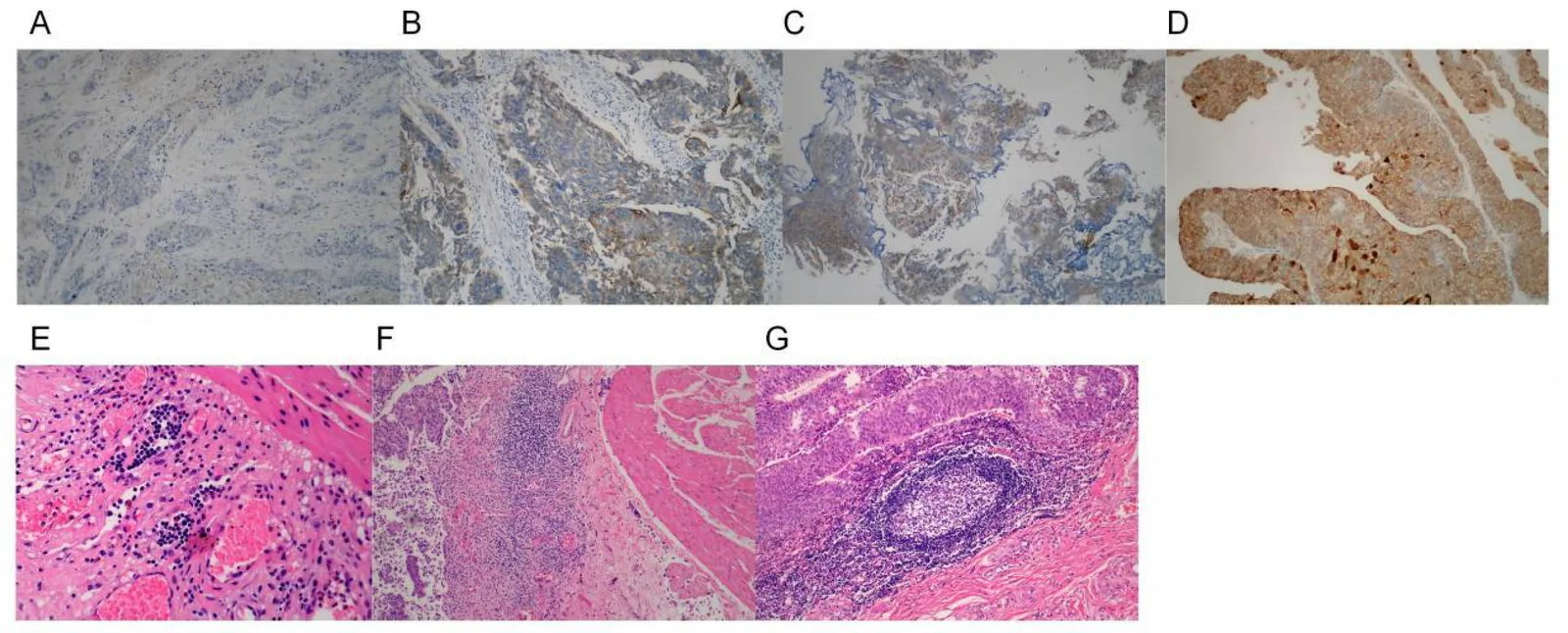
HER2 (**A-D** represent 0+, 1+, 2+, 3+ respectively) and TLS (**E-G** represent TLS-1, TLS-2, TLS-3 respectively) pathological images. Scale bars: 200 μm for **(A-F)** (×100 magnification); 100 μm for **(D, G)** (×200 magnification).

### Safety analysis

The overall incidence of TRAEs was 89.47%, with the vast majority being grade 1–2, suggesting generally good tolerability. A total of 5 cases (13.15%) experienced grade 3 TRAEs, all of which were effectively managed through dose adjustment or symptomatic treatment, and no grade 4 or 5 TRAEs were observed. The most frequently reported TRAEs included fatigue (34.21%), peripheral neuropathy (28.95%), anemia (26.32%), and pruritus (26.32%) (**Table 3**). Grade 3 TRAEs mainly involved peripheral neuropathy, anemia, leukopenia, and elevated transaminases. No treatment-related deaths occurred during the study period.

**Table 3:** Adverse events associated with neoadjuvant therapy.

Adverse events, n (%)	Patients (N = 38) Any grade	Grade ≥3
Asthenia	13 (34.21%)	0
Peripheral neuropathy	11 (28.95%)	2 (5.26%)
Anemia	10 (26.32%)	1 (2.63%)
Pruritus	10 (26.32%)	0
Nausea	9 (23.68%)	0
Leukopenia	8 (21.05%)	1 (2.63%)
Neutropenia	8 (21.05%)	0
Increased serum creatinine	7 (18.42%)	0
Elevated transaminases	6 (15.79%)	1 (2.63%)
Hypoalbuminemia	5 (13.16%)	0
Hyperglycemia	5 (13.16%)	0
Weight loss	3 (7.89%)	0
Thyroid dysfunction	1 (2.63%)	0

## Discussion

Muscle-invasive bladder cancer is one of the most aggressive malignancies of the urinary system, accounting for approximately 20%–25% of newly diagnosed bladder cancer cases but responsible for over 50% of bladder cancer-related deaths (17). Notably, about 30%–40% of patients with non-muscle-invasive bladder cancer may still progress to MIBC postoperatively, with a drastically reduced 5-year survival rate following progression (18, 19). For patients with cT2–4aN0M0 stage MIBC, neoadjuvant chemotherapy followed by RC has become the standard treatment modality. Cisplatin-based chemotherapy regimens can increase the pCR rate to 25%–35% and confer overall survival benefits (20, 21). However, nearly half of all patients are ineligible for cisplatin-based chemotherapy due to factors such as renal insufficiency, poor performance status, or comorbidities. Moreover, even after completing chemotherapy, a considerable proportion of patients still experience disease progression or recurrence (22, 23). Therefore, exploring novel neoadjuvant treatment strategies that are highly effective, low in toxicity, and applicable to a broader patient population has become a critical clinical challenge in the MIBC field.

RC48 is a HER2-targeted antibody–drug conjugate that specifically binds to HER2-positive tumor cells and releases monomethyl auristatin E (MMAE), inducing tumor cell apoptosis and immunogenic cell death (24, 25). RC48 has shown promising efficacy in previously treated patients with advanced urothelial carcinoma who have received multiple lines of therapy. A pooled analysis of two phase II studies (RC48-C005 and RC48-C009) reported an objective response rate (ORR) of 50.5% and a median overall survival (OS) of 14.2 months (26). Meanwhile, PD-1/PD-L1 immune checkpoint inhibitors (ICIs), which restore antitumor immune responses by blocking T-cell negative regulatory signals, have demonstrated significant activity in the neoadjuvant treatment of MIBC (27). The PURE-01 study revealed that pembrolizumab monotherapy achieved a pCR rate of 42% as neoadjuvant treatment (28). In the ABACUS study, atezolizumab showed a pCR rate of 31% (29). Based on the synergistic mechanism between ADCs and ICIs, ADC-induced tumor cell death promotes antigen release and enhances T-cell infiltration, whereas ICIs relieve immunosuppression, suggesting a potential synergistic effect when combined (30). An open-label phase II clinical study preliminarily confirmed that RC48 combined with toripalimab exhibited excellent efficacy in bladder-preserving therapy for MIBC, with 83.3% of patients achieving complete response, providing important evidence for the application of ADC–immunotherapy combinations in the MIBC field (31). Recent studies have demonstrated that TLS classification and maturation correlate with enhanced antitumor immunity and favorable prognosis in MIBC (32). In HER2-positive cancers, TLS has emerged as a promising prognostic biomarker, with high TLS expression linked to improved survival and better response to neoadjuvant therapy (33). Therefore, integrating HER2 expression with TLS status may optimize patient selection for RC48 plus PD-1 blockade, where HER2 determines targetability and TLS reflects immune fitness. This hypothesis warrants prospective validation in larger cohorts.

This multicenter retrospective real-world study included 38 patients with MIBC who received neoadjuvant therapy with RC48 combined with PD-1 inhibitors, all of whom subsequently underwent radical cystectomy. Previous studies have primarily focused on overall pathological complete response rates and general safety profiles, whereas the distinctiveness of this study is reflected in two aspects. One is the stratified analysis according to HER2 expression levels, which particularly reveals the marked disparity in therapeutic responses between the HER2 3+ subgroup and the HER2-low subgroup. The other is the preliminary exploration of higher TLS status as a potential modulator of treatment efficacy (12). The results demonstrated an CR rate of 36.8%, a pCR rate of 34.2%, a PR rate of 31.6%, a 12-month DFS rate of 89.4% (95% CI: 74.2%–95.9%), and a 12-month OS rate of 100%. We compared our findings with those from previously published literature on neoadjuvant chemotherapy. A large multicenter evaluation involving 600 patients reported a pCR rate of 22.1% for the gemcitabine–cisplatin (GC) regimen in MIBC patients (34). HER2 overexpression was significantly associated with higher pathological response rates (OR = 22.50, 95% CI: 1.51–335.33, P = 0.024), suggesting that HER2 expression levels may serve as a predictive variable for the efficacy of RC48 combined with immunotherapy. Stratified analysis based on TLS revealed that both primary and secondary lymphoid follicles were associated with significantly higher response rates compared with early lymphocyte aggregation (OR = 20.00 and 36.00, respectively; both P < 0.01), indicating that greater TLS correlates with enhanced clinical benefit from the combination therapy. Additionally, T3 stage, N1 stage, and N2 stage were each significantly associated with poorer treatment responses (all P < 0.05), suggesting that tumor burden and lymph node metastasis status remain key factors influencing the efficacy of neoadjuvant therapy. Regarding safety, TRAEs were generally manageable, and no treatment-related deaths were reported.

This study has several limitations. First, the retrospective design is subject to inherent biases, and the relatively small sample size limits the statistical power of the multivariate analysis, necessitating cautious interpretation of the generalizability of the findings. Second, heterogeneity existed across centers regarding the choice of PD-1 inhibitors, treatment cycles, and dose adjustments, which may introduce confounding effects on efficacy evaluation. The current sample size does not permit robust interaction analyses stratified by HER2 expression and TLS status across the two PD-1 inhibitors; however, we are actively expanding our patient cohort by incorporating data from additional centers and conducting a prospective multicenter study to enable more definitive subgroup analyses in the future. Third, the median follow-up duration of 18.0 months is relatively short, and long-term survival outcomes as well as delayed adverse events require further observation. We are actively extending follow-up for all enrolled patients and collecting long-term DFS and OS data, with the aim of reporting 3-year and longer outcomes in future updates, while also incorporating data from additional multicenter cohorts to enhance generalizability. Fourth, although TLS were evaluated independently by two experienced pathologists, immunohistochemical validation for TLS maturation markers, standardized whole-slide digital quantitation, and inter-observer Kappa agreement analysis were not performed; therefore, our H&E-based classification serves only as a morphological surrogate for TLS maturation status and is inherently subject to potential misclassification and inter-observer variability. Additionally, dynamic changes in the variables TLS and HER2, as well as their predictive value for treatment response, were not assessed due to the retrospective nature of specimen collection. To address this, we are systematically collecting paired pre- and post-treatment specimens to enable comprehensive evaluation of dynamic biomarker changes and their correlation with therapeutic efficacy. Furthermore, the absence of control groups receiving targeted therapy alone or immunotherapy alone precludes direct comparison of the incremental benefit of the combination therapy. We are actively collecting data from patients meeting criteria for cisplatin-based chemotherapy to enable comparative analyses between different neoadjuvant regimens in real-world settings.

## Conclusion

The combination of RC48 and a PD-1 inhibitor as a neoadjuvant treatment for MIBC demonstrates favorable pathological response rates and short-term survival benefits. HER2 expression levels and higher TLS status may potentially serve as stratification tools for identifying patients most likely to benefit from this regimen.

## Data Availability

The raw data supporting the conclusions of this article will be made available by the authors, without undue reservation.
