# Supplementary material for: Neoadjuvant disitamab vedotin (RC-48) plus a PD-1 inhibitor in muscle-invasive bladder cancer: a multicenter real-world study with stratification by HER2 expression and tertiary lymphoid structures
Source: Front Immunol. 2026 Sep 28;17:1891151. doi: 10.3389/fimmu.2026.1891151 (PMC13655032; doi:10.3389/fimmu.2026.1891151)
Supplement: Supplementary file 1 [file Table1.docx]

**Figure S1 Patient screening flow chart**

**Table S1 Subgroup analysis of drug types based on DFS**

| Variables | n (%) | RC48 + toripalimab | RC48 + tislelizumab | HR (95%CI) | *P* | P for interaction |
| --- | --- | --- | --- | --- | --- | --- |
|  |  |  |  |  |  |  |
| All patients | 38 (100.00) | 6/21 | 5/17 | 1.06 (0.32 ~ 3.48) | 0.922 |  |
| Age |  |  |  |  |  | 0.242 |
| ≤60 | 16 (42.11) | 2/6 | 2/10 | 0.51 (0.07 ~ 3.68) | 0.507 |  |
| >60 | 22 (57.89) | 4/15 | 3/7 | 2.13 (0.47 ~ 9.59) | 0.325 |  |
| Gender, n(%) |  |  |  |  |  | 0.222 |
| Female | 12 (31.58) | 1/6 | 3/6 | 3.05 (0.32 ~ 29.42) | 0.336 |  |
| Male | 26 (68.42) | 5/15 | 2/11 | 0.57 (0.11 ~ 2.94) | 0.502 |  |
| ECOG, n(%) |  |  |  |  |  | 0.997 |
| 0 | 25 (65.79) | 3/13 | 3/12 | 1.09 (0.22 ~ 5.39) | 0.918 |  |
| 1 | 13 (34.21) | 3/8 | 2/5 | 1.08 (0.18 ~ 6.50) | 0.929 |  |
| Lymphovascular invasion, n(%) |  |  |  |  |  | 0.986 |
| No | 24 (63.16) | 1/13 | 1/11 | 1.26 (0.08 ~ 20.24) | 0.868 |  |
| Yes | 14 (36.84) | 5/8 | 4/6 | 1.20 (0.32 ~ 4.58) | 0.786 |  |
| HR: Hazard Ratio, CI: Confidence Interval | | | | | | |

**Table S2 Subgroup analysis of drug types based on OS**

| Variables | n (%) | RC48 + toripalimab | RC48 + tislelizumab | HR (95%CI) | *P* | P for interaction |
| --- | --- | --- | --- | --- | --- | --- |
|  |  |  |  |  |  |  |
| All patients | 38 (100.00) | 5/21 | 5/17 | 1.79 (0.48 ~ 6.66) | 0.388 |  |
| Age |  |  |  |  |  | 0.206 |
| ≤60 | 16 (42.11) | 2/6 | 2/10 | 0.67 (0.09 ~ 4.85) | 0.696 |  |
| >60 | 22 (57.89) | 3/15 | 3/7 | 3.94 (0.65 ~ 23.82) | 0.135 |  |
| Gender, n(%) |  |  |  |  |  | 0.657 |
| Female | 12 (31.58) | 2/6 | 2/6 | 0.66 (0.09 ~ 4.88) | 0.683 |  |
| Male | 26 (68.42) | 3/15 | 3/11 | 2.60 (0.43 ~ 15.60) | 0.297 |  |
| ECOG, n(%) |  |  |  |  |  | 0.823 |
| 0 | 25 (65.79) | 3/13 | 3/12 | 1.40 (0.28 ~ 6.98) | 0.678 |  |
| 1 | 13 (34.21) | 2/8 | 2/5 | 3.16 (0.29 ~ 34.90) | 0.348 |  |
| Lymphovascular invasion, n(%) |  |  |  |  |  | 0.825 |
| No | 24 (63.16) | 1/13 | 1/11 | 1.39 (0.09 ~ 22.20) | 0.817 |  |
| Yes | 14 (36.84) | 4/8 | 4/6 | 2.24 (0.49 ~ 10.19) | 0.296 |  |
| N stage, n(%) |  |  |  |  |  | 0.421 |
| N0 | 17 (44.74) | 1/9 | 2/8 | 2.33 (0.21 ~ 25.73) | 0.490 |  |
| N1 | 16 (42.11) | 2/9 | 2/7 | 4.54 (0.39 ~ 52.78) | 0.226 |  |
| N2 | 5 (13.16) | 2/3 | 1/2 | 0.44 (0.04 ~ 5.13) | 0.512 |  |
| HR: Hazard Ratio, CI: Confidence Interval | | | | | | |

**Table S3 Pathologic Responses of Patients**

| Pathologic Responses | Patients (n = 38) |
| --- | --- |
| Pathological complete response (pCR), n (%) | 13 (34.2) |
| Pathological downstaging, n (%) | 31 (81.6) |
| **Pathological staging, n (%)** |  |
| ypT0N0 | 13 (34.2) |
| ypT0N+ | 1 (2.6) |
| ypT1N0 | 8 (21.1) |
| ypT1N+ | 2 (5.3) |
| ypT2N0 | 10 (26.3) |
| ypT2N+ | 2 (5.3) |
| ypT3-4N0 | 1 (2.6) |
| ypT3-4N+ | 1 (2.6) |

**Table S4 Treatment Delivery and Surgical Outcomes**

| Variables | Patients (n = 38) |
| --- | --- |
| **Treatment-related variables** |  |
| Neoadjuvant treatment cycles, median (range) | 5 (4–8) |
| Dose adjustments, n (%) | 5 (13.2) |
| Treatment delays (>7 days), n (%) | 8 (21.1) |
| Treatment discontinuation, n (%) | 2 (5.3) |
| Due to disease progression | 2 (5.3) |
| Due to toxicity | 0 (0.0) |
| Interval between last treatment and surgery, median (range), days | 25 (21–28) |
| **Surgical and pathological variables** |  |
| Surgical margin, n (%) |  |
| R0 (negative) | 38 (100) |
| R1 (positive) | 0 (0.0) |
| **Postoperative complications (Clavien-Dindo classification), n (%)** |  |
| Grade 0–I | 28 (73.7) |
| Grade II | 7 (18.4) |
| Grade III | 3 (7.9) |
| Grade IV–V | 0 (0.0) |
| **Adjuvant therapy after surgery, n (%)** |  |
| Yes | 6 (15.8) |
| No | 32 (84.2) |
| **Distribution of treatment cycles, n (%)** |  |
| 4 | 10 (26.3) |
| 5 | 10 (26.3) |
| 6 | 8 (21.1) |
| 7 | 6 (15.8) |
| 8 | 4 (10.5) |

**A**

**B**

**Figure S2 DFS (A) and OS (B) by PD-1 drug type**
